# Antiseptics and disinfectants for the treatment of bacterial vaginosis: A systematic review

**DOI:** 10.1186/1471-2334-12-148

**Published:** 2012-06-28

**Authors:** Hans Verstraelen, Rita Verhelst, Kristien Roelens, Marleen Temmerman

**Affiliations:** 1 Department of Obstetrics & Gynaecology, Faculty of Medicine and Health Sciences, Ghent University, De Pintelaan 185, B-9000, Ghent, Belgium; 2 International Centre or Reproductive Health, Faculty of Medicine and Health Sciences, Ghent University, Ghent, Belgium

**Keywords:** Bacterial vaginosis, Antiseptics, Disinfectants, Therapy, Systematic review, Chlorhexidine, Polyhexamethylene biguanide, Hydrogen peroxide

## Abstract

**Background:**

The study objective was to assess the available data on efficacy and tolerability of antiseptics and disinfectants in treating bacterial vaginosis (BV).

**Methods:**

A systematic search was conducted by consulting PubMed (1966-2010), CINAHL (1982-2010), IPA (1970-2010), and the Cochrane CENTRAL databases. Clinical trials were searched for by the generic names of all antiseptics and disinfectants listed in the Anatomical Therapeutic Chemical (ATC) Classification System under the code D08A. Clinical trials were considered eligible if the efficacy of antiseptics and disinfectants in the treatment of BV was assessed in comparison to placebo or standard antibiotic treatment with metronidazole or clindamycin and if diagnosis of BV relied on standard criteria such as Amsel’s and Nugent’s criteria.

**Results:**

A total of 262 articles were found, of which 15 reports on clinical trials were assessed. Of these, four randomised controlled trials (RCTs) were withheld from analysis. Reasons for exclusion were primarily the lack of standard criteria to diagnose BV or to assess cure, and control treatment not involving placebo or standard antibiotic treatment. Risk of bias for the included studies was assessed with the Cochrane Collaboration’s tool for assessing risk of bias. Three studies showed non-inferiority of chlorhexidine and polyhexamethylene biguanide compared to metronidazole or clindamycin. One RCT found that a single vaginal douche with hydrogen peroxide was slightly, though significantly less effective than a single oral dose of metronidazole.

**Conclusion:**

The use of antiseptics and disinfectants for the treatment of BV has been poorly studied and most studies are somehow methodologically flawed. There is insufficient evidence at present to advocate the use of these agents, although some studies suggest that some antiseptics may have equal efficacy compared to clindamycin or metronidazole. Further study is warranted with special regard to the long-term efficacy and safety of antiseptics and disinfectants for vaginal use.

## Background

Bacterial vaginosis (BV) is a condition characterised by the partial loss of the indigenous vaginal lactobacilli coupled with polymicrobial anaerobic overgrowth of the vaginal epithelium. Although BV often remains asymptomatic, it is one of the most common causes of vaginitis, and hence among the most common reasons for women to seek medical help [1] In recent years BV has further emerged as a global issue of concern due to its association with ascending genital tract infection and with sexually transmitted infections [2].

Current recommendations by the Centres for Disease Control and Prevention (CDC) for the treatment of BV basically involve antibiotic treatment with oral or intravaginal metronidazole or clindamycin [3]. Although these treatment modes are associated with fairly good short-term cure rates, they fail to prevent BV in at least half of the cases in the long run [4]. Several alternative treatment approaches are therefore being (re)considered, including the use of antiseptics and disinfectants.

Antiseptics have been used for over half a century in the treatment of vaginal infections. Similar to antibiotics, antiseptics facilitate the eradication of the anaerobic vaginal microbiota associated with bacterial vaginosis which allows for the recolonisation of indigenous lactobacilli. Antiseptics generally have a very broad spectrum as they act non-specifically on bacteria through mechanisms such as bacterial cell membrane disruption. In accordance, there are very few reports on antimicrobial resistance to these agents. Nonetheless, antiseptics and disinfectants have become unpopular for the purpose of treating vaginal infections, presumably because they are often used as over-the-counter (OTC) products associated with vaginal douching, a common practice that has been associated with the occurrence of BV. In many countries, antiseptics are still marketed for the treatment of vaginal infections, though their efficacy has not been supported by a sound evidence base. We therefore sought to assess currently available data on the efficacy and tolerability of antiseptics and disinfectants in the treatment of BV. Through a systematic literature search, we identified and evaluated clinical trials that compared antiseptic and/or disinfectant treatment to either placebo or standard antibiotic treatment in women of any age.

## Methods

### Objectives

The overall objective is to summarize currently available data (published up to December 31 2010) on the efficacy and tolerability of antiseptics and disinfectants in the treatment of BV, thereby accounting for the quality of the clinical trials identified through a systematic literature search.

### Search strategy

An electronic search was conducted by consulting the following databases: PubMed (1966-2010), CINAHL (1982-2010), International Pharmaceutical Abstracts (IPA) database (1970-2010), and the Cochrane Central Register of Controlled Trials.

Clinical trials were searched for by use of the key words “bacterial vaginosis” and “non-specific vaginitis” in combination with “disinfectant”, “antiseptic”, and subsequently with all generic names of antiseptics and disinfectants listed in the Anatomical Therapeutic Chemical (ATC) Classification System under the code D08A (Antiseptics and disinfectants), i.e. ethacridine lactate, aminoacridine, euflavine, aluminium agents, dibrompropamidine, chlorhexidine, propamidine, hexamidine, polyhexanide, boric acid, hexachlorophene, policresulen, phenol, triclosan, chloroxylenol, biphenylol, nitrofural, iodine/octylphenoxypolyglycolether, povidone-iodine, iodine, diiodohydroxypropane, dequalinium, chlorquinaldol, oxyquinoline, clioquinol, benzalkonium, cetrimonium, cetylpyridinium, cetrimide, benzoxonium chloride, didecyldimethylammonium chloride, benzethonium chloride, octenidine, benzethonium chloride, dodeclonium bromide, mercuric amidochloride, phenylmercuric borate, mercuric chloride, mercurochrome, mercury, thiomersal, mercuric iodide, phenylmercuric borate, silver nitrate, silver, hydrogen peroxide, eosin, propanol, tosylchloramide sodium, isopropanol, potassium permanganate, sodium hypochlorite, and ethanol.

In addition we accounted for benzydamine which is listed under ATC code G02CC03 for “anti-inflammatory products for vaginal administration”, considering benzydamine also has broad antimicrobial activity.

Only English-language studies were considered for review. Additional studies were searched for by checking cross-references cited in the primary studies. No efforts were made to identify unpublished studies.

### Types of studies

Reports were considered eligible if they involved clinical trials in which the efficacy and tolerability of antiseptics and disinfectants in the treatment of BV was assessed in comparison to placebo or in comparison to standard antibiotic treatment with metronidazole or clindamycin.

Studies were considered eligible if at least 40 study participants had been enrolled for comparison between the two treatment arms.

### Types of participants

Women of any age diagnosed with bacterial vaginosis through standardized criteria like Amsel’s or Nugent criteria [5,6]. No study was excluded because of (possible) co-infection with sexually transmitted infections.

### Types of intervention

We confined our review to antiseptics and disinfectants for which the dosage and treatment regimen was specified, and which are listed in the Anatomical Therapeutic Chemical (ATC) Classification System under the code D08A (“antiseptics and disinfectants”). In addition we accounted for benzydamine which is listed under ATC code G02CC03 for “anti-inflammatory products for vaginal administration”, considering benzydamine also has broad antimicrobial activity. Hence, we excluded any report in which any antiseptic product (e.g. herbal medicines) was used for which the chemical composition and/or substance dose was not verifiable. Treatment with an antiseptic or disinfectant was considered in any preparation type, any dosage regimen, and any route of administration.

### Types of outcome measures

The primary outcome was cure of bacterial vaginosis as assessed through use of the Amsel criteria or Nugent criteria at least 7 days following treatment initiation. Cure was defined as a Nugent score <7 or the presence of less than 3 Amsel criteria. The secondary outcome was the occurrence of adverse reactions or side effects, with special regard to vaginal symptoms following local administration of antiseptics.

### Risk of bias in included studies

As indicated by the PRISMA guidelines no efforts were made to label the quality of studies in a (semi-)quantitative matter [7]. Rather, the Cochrane Collaboration tool for assessing risk of bias was applied to assess the risk of various sources of bias among the included studies [8].

## Results

**Figure 1:**
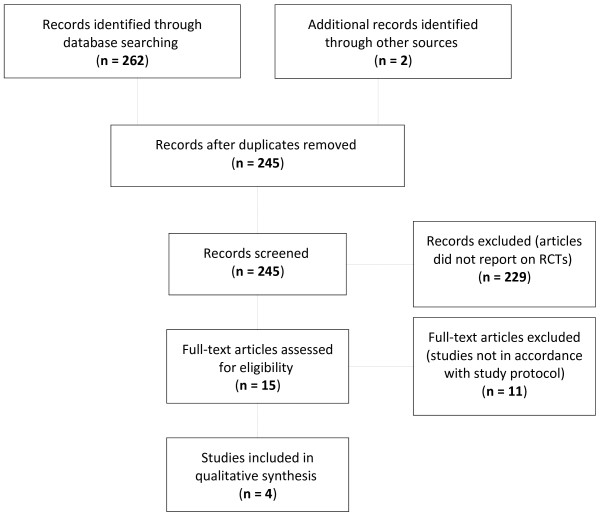
Literature search and selection process (PRISMA Flow Diagram).

Through the Boolean search of the electronic databases PubMed, CINAHL, IPA, and Cochrane CENTRAL, a total of 262 articles on BV and antiseptics and disinfectants were identified. Two additional articles were retrieved during the background literature study [9,10]. Eventually, 15 full-text papers were assessed for eligibility. Of these, four randomised controlled trials (RCTs) met the inclusion criteria set forth. The search and selection strategy is presented as a PRISMA flow diagram in Figure 1. 

The 15 clinical trials identified involved treatment with one or more of the following antiseptics and disinfectants: benzydamine (2 RCTs), chlorhexidine (2 RCTs), dequalinium chloride (1 RCT), hydrogen peroxide (3 clinical trials including 1 RCT), octenidine (1 RCT), polyhexamethylene biguanide (2 RCTs), and povidone iodine (6 clinical trials including 5 RCTs). These studies are herewith listed and briefly discussed according to the antiseptic or disinfectant under study.

### Benzydamine

We found two randomized clinical trials on treatment of BV with benzydamine of which none was in accordance with the inclusion criteria set forth [10,11].

In the 1987 double-blinded randomised controlled trial by Ventolini *et al.*[11] a regime of intravaginal 0.1% benzydamine hydrochloride applied twice daily for 10 days was compared nor to placebo, neither to standard antibiotic treatment. In addition no standard criteria were applied for the diagnosis of BV neither at recruitment nor at follow-up. Hence we considered this study not assessable for the purpose of the present study.

Hay *et al.* conducted a small double-blinded randomised controlled trial in which 15 patients were enrolled of which 8 used 140 ml of 0.1% benzydamine vaginal douche twice a day for 7 days and 7 patients applied a similar regimen with placebo douches [10]. Diagnosis of BV was made through Amsel criteria and Nugent score at enrolment and cure was defined as a normal Gram stain and absence of clue cells on wet mount. Since the sample size was below the lower limit set forth, we did not include the study.

### Chlorhexidine

We identified two single-blinded randomised controlled trials in which the efficacy of vaginally administered chlorhexidine with a chlorhexidine-containing pessary [12] and a chlorhexidine-based vaginal gel respectively [13] was compared to treatment of BV with metronidazole.

In the 1987 study by Ison *et al.*[12] 79 women attending an outpatient London clinic and diagnosed with BV according to Amsel’s criteria were enrolled, two thirds of which had a history of recurrent vaginosis. Women were randomised (randomisation procedure and allocation concealment not specified) to receive either a single pessary containing 150 mg chlorhexidine for two consecutive nights (n = 43), or a single 2 g oral dose of metronidazole (n = 36). Upon blinded reassessment after seven days, 84% of women in the chlorhexidine arm were found to be negative for all Amsel criteria, as were 78% of women in the metronidazole group (see Table 1). Sixty-three out of the 79 study participants were again evaluated in a blind manner at 28 days, and cure rates according to Amsel’s criteria were 52 and 60% in the chlorhexidine and metronidazole group (see Table 1), respectively. No mention is made of any adverse effects in this report. 

**Table 1:** Main characteristics of included studies

**Reference**	**Experimental treatment**	**Control treatment**	**Duration of follow-up**	**Method applied to assess cure**	**Cure rates**	**Risk ratio (95% CI, p-value)**
Ison 1987	pessary containing 150 mg **chlorhexidine** for 2 consecutive nights	single 2 g oral dose of **metronidazole**	7 days	Amsel criteria	36/43 versus 28/36	1.08 [0.86; 1.34], p = 0.5
			28 days		14/27 versus 15/25	1.16 [0.71; 1.88], p = 0.6
Molteni 2004	**chlorhexidine** vaginal gel (2.5 g chlorhexidine per dose) applied for seven days	500 mg **metronidazole** vaginal tablet daily for seven days	28 days	Amsel criteria	28/30 versus 11/15	0.79 [0.57; 1.08], p = 0.06
Chaithongwongwatthana 2003	a single **hydrogen peroxide** vaginal douche/placebo tablets similar to the metronidazole tablets	4 tablets of 500 mg **metronidazole**/placebo vaginal douche with clean water	14 days	Amsel criteria	45/72 versus 55/70	1.26[1.01; 1.56], p = .036
Minozzi 2008	single dose of 100 ml of a 10% **polyhexamethylene biguanide** vaginal gel solution	2% **clindamycin** cream for 7 days	21 to 30 days	Amsel criteria and Nugent score	153/175 versus 143/172 (Amsel)	0.95 [0.87; 1.04], p = 0.3
					99/175 versus 99/172 (Nugent)	1.02 [0.85; 1.22], p = 0.9

In the 2004 multicentre Italian study by Molteni *et al.*[13], 90 women were enrolled in four outpatient gynaecology services and one STD clinic, half of which who were diagnosed with *Candida* vaginitis, the other half being diagnosed with BV according to Amsel’s criteria. Women with BV (n = 45) were randomised according to a computer-generated randomisation scheme in a 2 to 1 ratio (allocation concealment not specified) with 30 women being assigned to the treatment group with chlorhexidine vaginal gel (2.5 g chlorhexidine per dose) applied for seven days and 15 women serving as controls taking a 500 mg metronidazole vaginal tablet daily for seven days. At week 4, blinded reassessment according to Amsel showed a cure rate of 93% in the chlorhexidine group compared to a 74% cure rate in the metronidazole group (see Table 1). With regard to side effects, Molteni *et al.* recorded that no serious adverse events were observed and that no women complained of vaginal discharge after treatment. In the experimental arm, overall six women (20%) complained of mild transient burning after chlorhexidine vaginal gel application.

### Dequalinium chloride

We found no controlled trials in which the efficacy of intravaginal dequalinium chloride in treating BV was compared to placebo or to standard antibiotic treatment. We did identify one large single-blinded, randomised controlled trial in which the efficacy of dequalinium chloride was weighed against that of povidone-iodine in treating BV [14].

### Hydrogen peroxide

We identified two non-controlled clinical trials that we excluded from this review [15,16], and one randomised triple-blinded controlled trial in which the efficacy of vaginal hydrogen peroxide instillation in curing BV was assessed [17].

In a very well-designed triple-blinded randomised controlled trial, Chaithongwongwatthana *et al.* investigated the effectiveness and safety of a single hydrogen peroxide vaginal douche in treating bacterial vaginosis [17]. In this study, 142 patients with BV were enrolled in a Thai outpatient gynaecologic clinic. Diagnosis was made according to Amsel’s criteria. Randomisation relied on computer-generated random number scheme and both subjects and investigators were blinded to treatment. In addition double dummies were used to ensure blinding. In the experimental treatment arm patients (n = 72) were treated with a single douche with 20 ml of a 3% hydrogen peroxide solution in lithotomy position for 3 minutes, whereas in the control arm patients (n = 70) received 4 tablets of 500 mg metronidazole. The dummy treatments consisted of a vaginal douche with clean water in the metronidazole group and of identical placebo tablets similar to the metronidazole tablets in the hydrogen peroxide group. Follow-up was performed at 2 weeks following treatment. Cure was defined as the absence of at least 3 Amsel criteria. Assessors were blinded for the treatment allocation. The cure rates were 62.5% in the hydrogen peroxide group and 78.6% in the metronidazole group (see Table 1). Patients in the metronidazole group had significantly more gastrointestinal side effects (48.6% versus 13.9%, p < 0.001), whereas patients in the hydrogen peroxide group had significantly more mild vaginal irritation (33.3% versus 14.3%, p = 0.008).

### Octenidine

In a large Serbian RCT, the efficacy of an octenidine hydrochloride/phenoxyethanol spray as compared to standard treatment with 500 mg metronidazole vaginal tablets for 7 days was assessed among 450 patients enrolled in an outpatient gynecology clinic and diagnosed with BV according to Amsel’s criteria [18]. Unfortunately, the authors did not specify *how* cure was defined. In particular, the authors state that study participants were *“*examined gynecologically” and that “control smears were taken for further bacteriologic analysis” [18]. We therefore repeatedly tried to contact the authors with a request for more detailed information; however we did not get any response. The otherwise very well designed study could therefore not be included.

### Polyhexamethylene biguanide

We identified two controlled clinical trials on treatment of BV with polyhexamethylene biguanide [19,20], a biguanide antiseptic that is actually listed in the Anatomical Therapeutic Chemical (ATC) Classification System as *polyhexanide*. One study was considered not assessable [19], whereas another large study was withheld [20].

In the 2003 single-blinded randomised controlled trial by Gerli *et al.*, 133 patients were enrolled of which 59 received a 2% polyhexamethylene biguanide gel solution and 51 administered 2% clindamycin cream for 7 days [19]. However, neither at enrolment nor at follow-up were all patients systematically assessed through Amsel’s or Nugent’s criteria and therefore we consider the study not assessable to the purpose of the present study.

The 2008 study by Minozzi *et al.* was a multi-centred single (investigator)-blinded randomised controlled trial in which 740 BV patients were enrolled [20]. Patients were considered eligible if they fulfilled all Amsel’s criteria and were then randomly assigned to one of two treatment arms, i.e. a single dose of 100 ml of a 10% polyhexamethylene biguanide vaginal gel solution (n = 371) or 2% clindamycin cream for 7 days (n = 369). Patients were then re-assessed through Amsel’s and Nugent’s criteria at 21 to 30 days following treatment initiation. Eventually data on 347 patients were evaluable; this is 46.9% of patients constituting the inception cohort. Hence, although attrition was high, the authors gave a detailed description for non-inclusion in the final per protocol analysis for 147 of the 196 non-included patients in the polyhexamethylene biguanide group and for of all 197 non-included patients in the clindamycin group. Cure was defined among others as the absence of all four, of three and of two of the Amsel’s criteria and was obtained in 64.3, 87.5, and 90.6% versus 63.2, 83.2, and 91.2% patients in the polyhexamethylene biguanide and clindamycin groups respectively (see Table 1). Reconversion to a normal microbiota (Nugent score <4) was achieved in 56.5 and 57.7% (see Table 1) of patients in the polyhexamethylene biguanide and clindamycin groups, while absence of clue cells was recorded in 87.5 and 84.0%, respectively. Thirty-seven patients in the polyhexamethylene biguanide group reported 29 study medication-related adverse events (AEs) compared to 29 patients in the clindamycin group who reported 22 study medication-related AEs (P = 0.3).

### Povidone iodine

We found six clinical trials in which povidone iodine was evaluated for its efficacy in the treatment of BV. One trial was a non-controlled study [21]. One study entailed a comparison with benzydamine [11], another study involved a comparison with dequalinium chloride [14], and a third one compared povidone iodine to a probiotic [22]. The remainder of studies were two placebo-controlled studies [9,23].

In the 1982 study by Dattani *et al.* patients were randomised to use either a 200 mg povidone-iodine containing pessary either an identical looking placebo pessary during night and morning for two weeks [23]. As no standard criteria (Amsel’s criteria or Nugent criteria) in this era were used either at inclusion or at follow-up, this study was discarded.

Van der Meijden *et al.*[9], also compared a 200 mg povidone-iodine containing pessary inserted into the vagina in the morning and evening for 5 consecutive days with placebo pessaries. As no standard criteria (Amsel’s criteria or Nugent criteria) in this era were used at inclusion or at follow-up, this study was therefore also considered not assessable.

In summary, of the 15 clinical trials identified through a systematic literature search, merely four RCTs with at least 40 study participants involved the comparison of a vaginal antiseptic to placebo or to standard antibiotic treatment and thereby relied on standard criteria in assessing BV and cure rates. Main characteristics of the latter studies are displayed in Table 1.

The four selected RCTs compared antiseptics to metronidazole or clindamycin. In two studies control treatment consisted of a single oral dose of 2 g metronidazole, in one study of a 7-day regime of 500 mg metronidazole intravaginally, and in one study of 2% clindamycin cream for 7 days. Only the latter two treatment modes are CDC recommended regimens [3]. Three studies showed non-inferiority of the antiseptics applied in comparison to antibiotic treatment. In one study, a single douche with 20 ml of a 3% hydrogen peroxide was significantly less effective than a single 2 g oral dose of metronidazole.

Only one RCT was double-blinded (and actually triple-blinded) through the use of double dummies [17]. Randomisation procedure was reported to involve a computer-generated random number series in two studies [13,17]. Allocation concealment was not mentioned explicitly in any study, except one [17]. As to selection bias, in none of the studies it was actually explicitly reported that consecutive patients were enrolled. In one large trial, attrition was very high albeit well-documented [20]. Follow-up ranged from 7 up to 28 days. In one out of the four RCTs no mention is made of recording of side effects [12]. Risk of selection, performance, detection, attrition, and reporting bias was assessed through use of the Cochrane Collaboration tool for assessing risk of bias [8] and is presented in Table 2. 

**Table 2:** Risk of bias for included studies

	**Ison 1987**	**Molteni 2004**	**Chaithongwongwatthana 2003**	**Minozzi 2008**
Random sequence generation	**?**	**+**	**+**	**?**
Allocation concealment	**?**	**?**	**+**	**?**
Blinding of participants and personnel	**-**	**-**	**+**	**-**
Blinding of outcome assessment	**+**	**+**	**+**	**?**
Incomplete outcome data	**-**	**+**	**+**	**-**
Selective reporting	**+**	**+**	**+**	**+**

***Legend.*+** indicates low risk of bias, **-** indicates high risk of bias, and **?** indicates unknown risk of bias.

With regard to side-effects, no mention is made of any adverse effects in the study on the chlorhexidine pessary [12]. In the study on chlorhexidine gel [13] it was merely mentioned that, overall, six women (20%) complained of mild transient burning after chlorhexidine vaginal gel application. In the hydrogen peroxide trial [17], patients in the metronidazole group had significantly more gastrointestinal side effects (48.6% versus 13.9%, p < 0.001), whereas patients in the hydrogen peroxide group had significantly more mild vaginal irritation (33.3% versus 14.3%, p = 0.008). Finally, in the polyhexamethylene biguanide study [20] it was reported that thirty-seven patients in the polyhexamethylene biguanide group (10.3%) reported 29 study medication-related adverse events (AEs) compared to 29 patients in the clindamycin group (7.9%) who reported 22 study medication-related AEs and that the number of patients who reported AEs and study medication-related AEs were not statistically different between the two groups (P = 0.386 and 0.336 respectively). A detailed overview of AEs was given in this report and no significant differences were observed for any given AE, including vaginal symptoms. So, overall in two of the three studies that reported AEs elevated rates of vaginal irritation or burning were recorded.

## Discussion

We sought to assess and summarize currently available data on the efficacy and tolerability of antiseptics and disinfectants in treating BV. Overall, we identified 15 clinical trials that dealt with treatment of BV with antiseptics and disinfectants. Taking into account the limitations of the studies, it was found that some of the antiseptics tested, i.e. chlorhexidine and polyhexamethylene biguanide were equally effective to antibiotic treatment, whereas in one study, a single douche of hydrogen peroxide was slightly, though significantly less effective than a single 2 g oral dose of metronidazole.

Eleven studies were discarded, because the studies had no RCT design, because diagnosis of BV at inclusion and/or on follow-up did not rely on standard criteria like Amsel’s or Nugent’s criteria, or because the control treatment arm did not involve placebo or standard antibiotic treatment.

Of the four studies included, only one RCT was double-blinded (and actually triple-blinded) through the use of double dummies. Risk of selection bias and of performance bias was generally high. Risk of attrition bias was high in one study. Reporting bias was presumably low. It should be added that the risk of publication bias is also presumably high, especially since published reports often involved commercial antiseptic products marketed for vaginal use.

Follow-up in all studies was limited, as has previously been noted with antibiotic treatment studies on BV [4]. Hence, it remains unclear at present whether antiseptics and disinfectants might offer an alternative to the poor long-term cure rates observed with clindamycin and metronidazole. On the other hand, antiseptics and disinfectants might be better suited for repeated treatment courses than antibiotics, one reason being the odds of antibacterial resistance occurring, as has been observed with clindamycin for instance [24,25].

Another issue of concern that has been insufficiently addressed at present is the safety of the antiseptic products and their excipients used. While one of the four studies we withheld for qualitative analysis did not make any mention of side effects, two of the three studies that reported adverse events reported increased rates of vaginal irritation or burning.

Though some agents like chlorhexidine and povidone iodine have been widely used for preoperative wound prophylaxis and have also been studied for perpartum GBS prophylaxis and as microbicides, we believe that the safety of these agents especially upon repeated use warrants further scrutiny. This is particularly important considering in many parts of the world women with BV are often also at elevated risk for HIV acquisition and since HIV infection may be enhanced when epithelial integrity is threatened by local products.

## Conclusions

It may be concluded that the use of antiseptics and disinfectants for the treatment of BV has been poorly studied and that most studies that did address the subject are somehow methodologically flawed. Limited data indicate that some antiseptics may have equal efficacy in treating BV on short term compared to treatment with clindamycin or metronidazole. Further study is however warranted with special regard to long-term efficacy and safety of antiseptics and disinfectants for vaginal use.

## Competing interests

The authors declare that they have no competing interests.

## Authors' Contributions

HV conceived of the study, collected the literature, assessed the data and drafted the manuscript. RV helped to draft the manuscript. KR assessed the data and helped to draft the manuscript. MT helped to draft the manuscript. All authors read and approved the final manuscript.

## Pre-publication history

The pre-publication history for this paper can be accessed here:

http://www.biomedcentral.com/1471-2334/12/148/prepub
